# Prevalence and prognostic value of baseline and post-treatment renal insufficiency in colorectal cancer patients: a large retrospective cohort study of 10,581 patients in Shanghai, China (2019–2023)

**DOI:** 10.3389/fmed.2025.1671990

**Published:** 2026-01-13

**Authors:** Juan Li, Chen Guang Bai, Baoshuai Liu, Shouyu Pan, Lianjie Liu, Jianjun Jiang, Jian Lu, Guangwen Cao, Wei Zhang, Xian Hua Gao

**Affiliations:** 1 Department of Nephrology, Changhai Hospital, Naval Medical University, Shanghai, China; 2 Department of Pathology, Changhai Hospital, Naval Medical University, Shanghai, China; 3 Department of Colorectal Surgery, Changhai Hospital, Naval Medical University, Shanghai, China; 4 Hereditary Colorectal Cancer Center and Genetic Block Center of Familial Cancer, Changhai Hospital, Shanghai, China; 5 Department of Anorectal Surgery, Kongjiang Hospital, Shanghai, China; 6 Department of Epidemiology, Naval Medical University, Shanghai, China

**Keywords:** chronic kidney disease, colorectal cancer, hematuria, proteinuria, renal insufficiency

## Abstract

**Background:**

Baseline renal insufficiency (RI) and post-treatment renal insufficiency (PTRI) are prevalent comorbidities in patients with colorectal cancer (CRC). However, the prevalence, risk factors, and prognostic significance of these conditions remain controversial.

**Methods:**

This is a retrospective cohort study, which included all CRC patients treated at Shanghai Changhai Hospital (2019–2023). Multivariate logistic and Cox survival analyses were used to explore risk factors and prognostic value of baseline RI and PTRI.

**Results:**

Among 10,581 CRC patients, the prevalence rates of RI-related parameters were: baseline RI (32.4%), hematuria (31.2%), elevated serum cystatin C (CysC) (17.4%), proteinuria (12.3%), elevated serum uric acid (8.7%), elevated serum creatinine (4.3%), elevated blood urea nitrogen (BUN) (3.5%), reduced estimated glomerular filtration rate (eGFR) (2.2%), chronic kidney disease (CKD) diagnosed by ultrasonography(1.5%), and self-reported CKD (1.1%). Approximately half of RI cases (47.2%) were attributed to hematuria, and other RI-related parameters were complementary to hematuria in revealing RI. Among the 2,132 (20.3%) patients with PTRI, 14.8, 8.3, 4.4, 3.9 and 0.2% exhibited a ≥ 25% alteration in BUN, CysC, creatinine, eGFR and UA, respectively. Age ≥ 65 years and concomitant cardiovascular disease were independent predictors of baseline RI; open surgery, intestinal ostomy, postoperative complications and chemoradiotherapy were independent predictors of PTRI. Both baseline RI and PTRI were independent predictors of disease-free survival and overall survival.

**Conclusion:**

These results demonstrate that comprehensive RI assessment (incorporating eGFR, hematuria, proteinuria, and other RI-related parameters) provides clinically actionable insights. Given the high prevalence (baseline RI 32.4%, PTRI 20.3%) and significant survival impacts, early screening of RI and preventive strategies are critical for high-risk CRC patients.

## 1 Introduction

Colorectal cancer (CRC) is a prevalent malignant tumor, with approximately one million new cases diagnosed annually worldwide. As the third most common malignant tumor and the second leading cause of cancer-related deaths in the United States, CRC accounted for approximately 153,020 new cases and 52,550 deaths in 2023 (1). CRC is also a significant public health concern in China. According to the latest nationwide cancer report from the National Cancer Center of China published in 2022, CRC ranks as the second most prevalent malignant tumor and the fourth most common cause of cancer-related deaths in China, with 408,000 new CRC cases and 195,600 CRC-related deaths (2).

Chronic kidney disease (CKD) is usually defined as the presence of an estimated glomerular filtration rate (eGFR) < 60 mL/min/1.73 m^2^, albuminuria [urine albumin-to-creatinine ratio (ACR) > 30 mg/g], or any other types of structural/functional renal abnormalities lasting for more than 3 months, regardless of the cause (3, 4). Globally, CKD prevalence has increased in recent decades. The prevalence of moderate renal insufficiency (RI) was 5.4% in the general population in the USA between 1988 and 1994, whereas it increased to 7.7% between 1999 and 2004 (5), and the prevalence of severe RI increased from 0.21 to 0.35% during the same period (5). In 2017, 1.2 million individuals succumbed to CKD globally, and CKD mortality rate increased by 41.4% between 1999 and 2017 (6). Global CKD prevalence in 2017 was estimated to be 9.1% in the general population (6), with approximately 15% of the population in the USA having CKD, and approximately 37 million Americans living with CKD in 2022 (7). A survey of 47,204 participants in China reported a CKD prevalence of 10.8% in the general population in 2009 (8). A national cross-sectional study conducted between 2018 and 2019, which included 176,874 adults in mainland China, demonstrated that the estimated prevalence of CKD, reduced eGFR, and albuminuria were 8.2, 2.2, and 6.7% in the general population, respectively (3). CKD is a severe disease that heightens the risk of cardiovascular disease and various other morbidities, potentially resulting in diminished quality of life and a substantial increase in mortality.

CKD is reported to be present in approximately 12–38% of patients with cancer. However, in a few studies with relatively small sample sizes, CKD prevalence in patients with CRC was documented to range from 2.4 to 26.8% (4, 9–11). In some instances, patients with CRC may already have pre-existing renal disease, and RI develops independently of CRC. For other patients, RI may be directly caused by the paraneoplastic syndrome of CRC or indirectly linked to adverse effects from cancer treatments. Various treatments of CRC, including surgery, chemoradiotherapy and targeted therapy, would lead to post-treatment renal insufficiency (PTRI). Moreover, preoperative baseline RI and PTRI can increase cardiovascular morbidity and mortality, impact drug tolerance, and potentially result in reduced perioperative chemoradiotherapy and an unfavorable prognosis (12–14). Conversely, patients with RI have a significantly elevated CRC risk compared with the general population (15, 16), and the prognostic value of PTRI remains inconclusive (17).

The exact prevalence, risk factors, and prognostic value of RI in patients with CRC remain inconclusive. Additionally, CKD is usually defined as RI lasting for more than 3 months, but determining the duration of RI in a retrospective study is difficult. Therefore, the terms preoperative baseline RI and PTRI were selected to replace CKD. An assessment of baseline RI and PTRI prevalence in a large cohort of CRC patients will facilitate an accurate evaluation and enhance the management and prognosis of this condition. This large retrospective cohort study aimed to clarify the prevalence, risk factors and prognostic value of baseline RI and PTRI in patients with CRC.

## 2 Materials and methods

This retrospective cohort study included all patients with CRC who underwent surgical resection at Changhai Hospital from January 2019 to December 2023. This study received approval from the Ethics Committee of Shanghai Changhai Hospital (B2019–008). Given the retrospective nature of this study, the Ethics Committee waived the need for written informed consent.

### 2.1 Inclusion and exclusion criteria

#### 2.1.1 Inclusion criteria

(1)    Patients with pathologically confirmed colorectal adenocarcinoma.
(2)    Patients with at least one RI-related parameter recorded in the electronic medical system database of Changhai Hospital.
(3)    Patients with CRC who underwent surgical resection from January 2019 to December 2023 at Changhai Hospital.

#### 2.1.2 Exclusion criteria

(1)    Patients with other pathological types of colorectal tumors, including colorectal lymphomas, neuroendocrine tumors, leiomyomas, and stromal tumors.
(2)    Patients with colorectal lesions invaded by malignant tumors from adjacent organs, such as gastric, pancreatic, ovarian, and uterine tumors.
(3)    Patients with colorectal lesions metastasized from malignant tumors of distant organs.
(4)    Patients with recurrent CRC.
(5)    Patients with a preoperative diagnosis of colorectal adenocarcinoma, but confirmed to have colorectal adenoma without adenocarcinoma by postoperative pathology.

### 2.2 Parameters and database

All parameters analyzed in this study were extracted from the following three databases.

(1)    The CRC database of the Colorectal Surgery Department at Changhai Hospital: It was established in 2000. Demographic characteristics, body weight, body height, body mass index (BMI), comorbidities, family history of cancer, clinicopathological features, treatment, and prognosis-related parameters, were recorded at admission and during follow-up.
(2)    The pathology registry: It is the authoritative record for all patients who undergo pathological examinations at our hospital. This database includes demographic data, tumor position, sampling date, pathological and molecular features.
(3)    The electronic medical system database of Changhai Hospital: It stores clinical and laboratory data, and ultrasonography examination results for both inpatients and outpatients. Extracted parameters from this database include results of: ➀ urinalysis (proteinuria, hematuria, concentration of urine protein and hemoglobin, and red blood cell count in the random urine); ➁ blood cell test (cell count of white blood cell [WBC], neutrophil, lymphocyte and platelet, and concentration of blood hemoglobin); ➂ kidney function related blood parameters [baseline and peak serum levels of creatinine, blood urea nitrogen (BUN), uric acid (UA), cystatin C (CysC), total protein, and albumin]; and ➃ ultrasonography of kidney.

### 2.3 Definitions of preoperative baseline RI, PTRI, and related parameters

Preoperative baseline RI was defined as the presence of any one of the following kidney structural or functional abnormalities: (1) eGFR < 60 mL/min/1.73 m^2^, (2) proteinuria; (3) hematuria or elevated urine hemoglobin; (4) elevated baseline serum levels of creatinine, BUN, UA, or CysC; (5) self-reported history of CKD; and (6) kidney with classical ultrasonography signs of CKD (18). The normal reference range for serum CysC is sex-specific: 0.63–1.25 μg/mL in males and 0.54–1.15 μg/mL in females. PTRI was defined as an increase in the peak serum levels of post-treatment creatinine, BUN, UA, or CysC from the corresponding baseline serum levels by ≥ 25%, or a decrease in the lowest post-treatment eGFR from the baseline eGFR by ≥ 25%.

Serum levels of baseline RI-related parameters were measured at the time of CRC diagnosis and repeated several times during treatment and follow-up. For parameters with repeated results, only two results were recorded and analyzed: the baseline serum level at CRC diagnosis and the peak serum level. Accordingly, the baseline and lowest eGFR were calculated using baseline and peak serum creatinine levels, respectively. In this study, eGFR was calculated from the serum level of creatinine (Scr) using the modified Modification of Diet in Renal Disease formula: eGFR = 175 × Scr^–1.234^ × age^–0.179^ [if female, × 0.79], where Scr was in mg/dL and age was in years (8). Patients were classified into five stages according to well-established staging system based on eGFR (9, 19): stage 1: eGFR ≥ 90 mL/min/1.73 m^2^; stage 2: eGFR between 60 and 89 mL/min/1.73 m^2^; stage 3: eGFR between 30 and 59 mL/min/1.73 m^2^; stage 4: eGFR between 15 and 29 mL/min/1.73 m^2^; and stage 5: eGFR < 15 mL/min/1.73 m^2^ (9, 19). Normal eGFR was defined as eGFR ≥ 60 mL/min/1.73 m^2^, and reduced eGFR was defined as eGFR < 60 mL/min/1.73 m^2^ (18).

Urinalysis included qualitative dipstick tests to detect red blood cell (RBC), hemoglobin, and protein levels. Urinalysis was performed at the diagnosis of CRC using fresh midstream urine samples. Positive primary screening results led to microscopic examination of the urinary sediment. Proteinuria was defined as a positive dipstick test for albumin or protein concentration > 0.15 g/L (20, 21). Hematuria was defined as a positive dipstick test for blood, or > 3 RBC per high power field (HP) (20), or urine hemoglobin > 3 g/L (or > 10 RBC/μL). The reported prevalence of hematuria and proteinuria were calculated exclusively among the tested sub-cohort. That is, only among patients who actually underwent urinalysis. A history of CKD was collected using a self-reported questionnaire. All patients with CRC were recommended to receive routine bilateral kidney ultrasonography. A patient was considered to have “CKD diagnosed by ultrasonography,” if the kidney displayed any of the following classic ultrasonography signs of CKD (22, 23): (1) smaller kidney size, (2) parenchyma thinning, (3) parenchyma hyperechogenicity, (4) unclear boundary between kidney cortex and medulla.

Cardiovascular disease encompasses a wide range of conditions affecting the heart and blood vessels, including coronary artery disease, myocardial infarction, heart failure, heart valve problems, arrhythmias, congenital heart disease, rheumatic heart disease, aortic disease, and peripheral artery disease. Cerebrovascular diseases are a group of conditions that affect the blood vessels in the brain, including stroke, brain aneurysm, brain bleed and carotid artery disease.

### 2.4 Statistical analysis

Statistical analyses were conducted using SPSS (version 22.0; IBM, Armonk, NY, United States). Continuous variables were described as means ± standard deviations (SD) or medians (interquartile ranges), while categorical variables are presented as frequency (percentage). Intergroup comparisons were performed using Student’s *t*-test for continuous variables and chi-square (χ^2^) test for categorical variables. Survival outcomes, including overall survival (OS) and disease-free survival (DFS), were analyzed through Kaplan-Meier methodology with log-rank testing, with time to event defined from the time of diagnosis until the end of follow-up (December 31, 2024) or date of death. The median follow-up time for the 10,581 CRC patients in our study was 29 months (interquartile range: 13–43 months). Patients who were lost to follow-up were censored at the date of their last known clinical or administrative contact. Loss to follow-up was defined as the absence of any such contact for more than 12 months in the absence of documented death. Overall, 8.2% of patients (*n* = 865) were lost to follow-up. In the OS analysis, all-cause mortality was treated as an event. In the DFS analysis, recurrence, metastasis and mortality were all treated as events, in accordance with standard analytical practice. Treatment interruptions—whether due to toxicity, patient preference, logistical challenges, or other reasons—were not considered events. A patient was classified as having received treatment if he had completed at least one cycle of the planned regimen. Multivariate logistic analysis was utilized to identify predictors of baseline RI and PTRI, whereas multivariate Cox survival analysis was used to determine predictors of OS and DFS using the forward stepwise model (likelihood ratio) with inclusion criterion of *P* < 0.05 and exclusion criterion of *P* > 0.10. The covariates included in our multivariable analyses were selected based on a combination of clinical relevance, prior literature on CRC outcomes, and statistical considerations. Specifically, we prespecified a set of variables known to be associated with both the exposure of interest and the outcomes, including age, sex, comorbidities (such as hypertension, diabetes, and cardiovascular disease), tumor characteristics (such as TNM stage, differentiation, vascular invasion, perineural invasion, tumor budding, tumor deposit, molecular feature, tumor markers), and treatment modality (such as radical resection, laparoscopic surgery, extensive resection, resection margin, intestinal ostomy, chemotherapy, radiotherapy, or multimodal therapy). All statistical tests were two-sided with α = 0.05 defining significance.

## 3 Results

### 3.1 Demographic characteristics and CKD stage of patients with CRC

**FIGURE 1:**
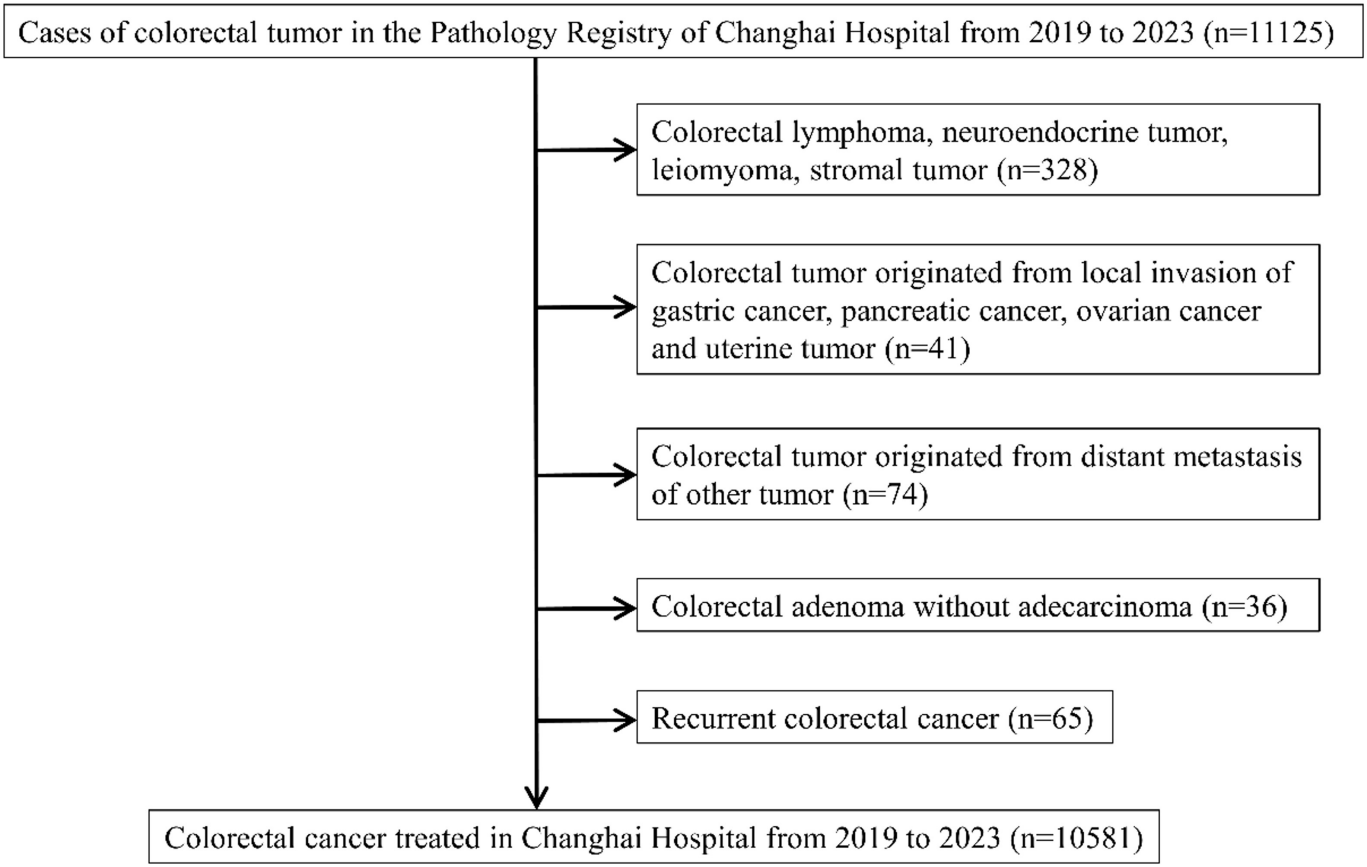
Flowchart of 21patient selection.

In total, 10,581 cases with pathologically confirmed CRC were included in this study. A flowchart illustrating the patient selection process is shown in Figure 1. Of the 10,581 patients, 63.8% were male and 36.2% were female (Table 1). Colon cancer accounted for 49.0% of cases, while rectal cancer accounted for 51.0% (Table 1). The prevalence of various comorbidities included hypertension (31.3%), cardiovascular disease (21.8%), diabetes (13.8%), CKD diagnosed using ultrasonography (1.5%), cerebrovascular disease (1.3%), respiratory disease (1.2%), and self-reported CKD (1.1%) (Table 1). The average age at diagnosis was 61.7 ± 11.5 years, and the mean BMI was 23.7 ± 4.7 kg/m^2^ (Table 2). According to baseline eGFR, 8,145 patients (77.7%) were classified as stage 1 CKD, 2,112 (20.1%) as stage 2, 213 (2.0%) as stage 3, 3 (0.1%) as stage 4, and 1 (0.0%) as stage 5 (Supplementary Table 1). According to the lowest eGFR, 7,337 patients (70.2%) were classified as stage 1 CKD, 2,734 (26.1%) as stage 2, 340 (3.3%) as stage 3, 45 (0.4%) as stage 4, and 2 (0.0%) as stage 5 (Supplementary Table 1).

**TABLE 1:** Relationship between preoperative baseline renal insufficiency (RI) and categorical parameters in patients with colorectal cancer.

Parameters		Patients without baseline RI (*N* = 7,154)	Patients with baseline RI (*N* = 3,427)	Total (*N* = 10,581)	*P* *
Sex	Male	4,584 (64.1%)	2,163 (63.1%)	6,747 (63.8%)	0.337
	Female	2,570 (35.9%)	1,264 (36.9%)	3,834 (36.2%)	
History of cancer	No	6,968 (97.4%)	3,325 (97.0%)	10,293 (97.3%)	0.265
	Yes	186 (2.6%)	102 (3.0%)	288 (2.7%)	
Cardiovascular disease	No	5,744 (80.3%)	2,534 (73.9%)	8,278 (78.2%)	** < 0.001**
	Yes	1,410 (19.7%)	893 (26.1%)	2,303 (21.8%)	
Hypertension	No	5,147 (71.9%)	2,125 (62.0%)	7,272 (68.7%)	** < 0.001**
	Yes	2,007 (28.1%)	1,302 (38.0%)	3,309 (31.3%)	
Cerebrovascular disease	No	7,078 (98.9%)	3,368 (98.3%)	10,446 (98.7%)	**0.005**
	Yes	76 (1.1%)	59 (1.7%)	135 (1.3%)	
Diabetes	No	6,216 (86.9%)	2,906 (84.8%)	9,122 (86.2%)	**0.004**
	Yes	938 (13.1%)	521 (15.2%)	1,459 (13.8%)	
Respiratory disease	No	7,077 (98.9%)	3,377 (98.5%)	10,454 (98.8%)	0.091
	Yes	77 (1.1%)	50 (1.5%)	127 (1.2%)	
Self-reported CKD*	No	7,154 (100.0%)	3,310 (96.6%)	10,464 (98.9%)	** < 0.001**
	Yes	0 (0.0%)	117 (3.4%)	117 (1.1%)	
CKD diagnosed by ultrasonography	No	4,156 (100.0%)	2,205 (95.7%)	6,361 (98.5%)	** < 0.001**
	Yes	0 (0.0%)	99 (4.3%)	99 (1.5%)	
Proteinuria	Negative	2,503 (100.0%)	2,053 (76.3%)	4,556 (87.7%)	** < 0.001**
	Positive	0 (0.0%)	638 (23.7%)	638 (12.3%)	
Hematuria	Negative	2,489 (100.0%)	1,086 (40.1%)	3,575 (68.8%)	** < 0.001**
	Positive	0 (0.0%)	1,619 (59.9%)	1,619 (31.2%)	
Baseline eGFR*	Normal	7,063 (100.0%)	3,194 (93.4%)	10,257 (97.8%)	** < 0.001**
	Reduced	0 (0.0%)	227 (6.6%)	227 (2.2%)	
Baseline serum creatinine	Normal	7,063 (100.0%)	2,965 (86.7%)	10,028 (95.7%)	** < 0.001**
	Elevated	0 (0.0%)	456 (13.3%)	456 (4.3%)	
Baseline serum BUN*	Normal	7,066 (100.0%)	3,051 (89.2%)	10,117 (96.5%)	** < 0.001**
	Elevated	0 (0.0%)	370 (10.8%)	370 (3.5%)	
Baseline serum UA*	Normal	7,067 (100.0%)	2,504 (73.2%)	9,571 (91.3%)	** < 0.001**
	Elevated	0 (0.0%)	917 (26.8%)	917 (8.7%)	
Baseline serum CysC*	Normal	3,636 (100.0%)	1,343 (56.1%)	4,979 (82.6%)	** < 0.001**
	Elevated	0 (0.0%)	1,050 (43.9%)	1,050 (17.4%)	
Tumor position	Colon	3,533 (49.4%)	1,647 (48.1%)	5,180 (49%)	0.202
	Rectum	3,621 (50.6%)	1,780 (51.9%)	5,401 (51%)	
TNM stage	I	1,121 (18.2%)	552 (18.5%)	1,673 (18.3%)	0.838
	II	2,163 (35.0%)	1,020 (34.2%)	3,183 (34.8%)	
	III	2,305 (37.3%)	1,116 (37.4%)	3,421 (37.4%)	
	IV	585 (9.5%)	294 (9.9%)	879 (9.6%)	
Differentiation	Well	49 (0.8%)	29 (1.0%)	78 (0.9%)	0.276
	Moderate	5,112 (82.7%)	2,453 (83.6%)	7,565 (83.0%)	
	Poor	1,018 (16.5%)	451 (15.4%)	1,469 (16.1%)	

*CKD, chronic kidney disease; eGFR, estimated glomerular filtration rate; reduced baseline eGFR was defined as baseline eGFR < 60 mL/min/1.73 m^2^; BUN, blood urea nitrogen; UA, uric acid; CysC, cystatin C. Bold values indicate statistical significance (*P* < 0.05).

**TABLE 2:** Relationship between preoperative baseline renal insufficiency (RI) and continuous parameters in patients with colorectal cancer.

Parameters	Patients without baseline RI (*N* = 7,154)	Patients with baseline RI (*N* = 3,427)	Total (*N* = 10,581)	*P* *
Age (year)	60.9 ± 11.3	63.4 ± 11.7	61.7 ± 11.5	** < 0.001**
	*N* = 7,154	*N* = 3,427	*N* = 10,581	
Height (cm)	165.7 ± 8.4	165.5 ± 7.8	165.7 ± 8.2	0.483
	*N* = 4,271	*N* = 2,022	*N* = 6,293	
Weight (kg)	64.8 ± 11.3	65.3 ± 11.7	65 ± 11.4	0.114
	*N* = 4,273	*N* = 2,022	*N* = 6,295	
Body mass index (kg/m^2^)	23.6 ± 5.2	23.7 ± 3.3	23.7 ± 4.7	0.391
	*N* = 4,271	*N* = 2,022	*N* = 6,293	
Baseline eGFR (mL/min/1.73 m^2^)*	115.3 ± 26.2	97.8 ± 28.0	109.6 ± 28.0	** < 0.001**
	*N* = 7,063	*N* = 3,421	*N* = 10,484	
Baseline serum creatinine (μmol/L)	66.7 ± 13.7	78.2 ± 25.1	70.4 ± 19.0	** < 0.001**
	*N* = 7,063	*N* = 3,421	*N* = 10,484	
Baseline serum BUN (mmol/L)*	4.3 ± 1.5	5.6 ± 2.1	4.7 ± 1.8	** < 0.001**
	*N* = 7,066	*N* = 3,421	*N* = 10,487	
Baseline serum UA (umol/L)*	232.1 ± 104.2	299.1 ± 142.9	254 ± 122.3	** < 0.001**
	*N* = 7,067	*N* = 3,421	*N* = 10,488	
Baseline serum CysC (mg/L)*	0.9 ± 0.2	1.1 ± 0.4	1.0 ± 0.3	** < 0.001**
	*N* = 3,636	*N* = 2,393	*N* = 6,029	
Serum level of total protein (g/L)	72.7 ± 8.7	71.2 ± 8.9	72.2 ± 8.8	0.176
	*N* = 7,063	*N* = 3,421	*N* = 10,484	
Serum level of albumin (g/L)	40.3 ± 4.7	39.4 ± 5.2	40.0 ± 4.9	0.268
	*N* = 7,063	*N* = 3,421	*N* = 10,484	
White blood cell (10^∧^9/L)	8.7 ± 4.3	7.1 ± 3.4	8.2 ± 4.1	** < 0.001**
	*N* = 7,097	*N* = 3,425	*N* = 10,522	
Neutrophil (10^∧^9/L)	6.6 ± 4.4	4.9 ± 3.3	6.1 ± 4.1	** < 0.001**
	*N* = 7,096	*N* = 3,425	*N* = 10,521	
Lymphocyte (10^∧^9/L)	1.3 ± 0.6	1.5 ± 0.7	1.4 ± 0.7	** < 0.001**
	*N* = 7,096	*N* = 3,425	*N* = 10,521	
Hemoglobin (g/L)	123.0 ± 20.9	123.7 ± 21.7	123.2 ± 21.2	0.149
	*N* = 7,097	*N* = 3,425	*N* = 10,522	
Platelet (10^∧^9/L)	223.0 ± 77.0	223.9 ± 77.5	223.3 ± 77.2	0.563
	*N* = 7,097	*N* = 3,425	*N* = 10,522	
Preoperative chemotherapy cycles	3.5 ± 2.2	3.5 ± 2.6	3.5 ± 2.4	0.994
	*N* = 411	*N* = 218	*N* = 629	
Preoperative hospital stay (day)	3.0 ± 2.1	3.7 ± 2.6	3.2 ± 2.3	** < 0.001**
	*N* = 4,137	*N* = 1,964	*N* = 6,101	
Postoperative hospital stay (day)	6.4 ± 6.7	7.4 ± 8.7	6.7 ± 7.4	** < 0.001**
	*N* = 4,135	*N* = 1,964	*N* = 6,099	
Length of hospital stay (day)	9.5 ± 3.5	11.0 ± 5.9	10.0 ± 4.5	** < 0.001**
	*N* = 4,222	*N* = 1,997	*N* = 6,219	
Postoperative chemotherapy cycles	5.4 ± 2.4	5.5 ± 2.5	5.4 ± 2.4	0.058
	*N* = 2,616	*N* = 1,042	*N* = 3,658	

*CKD, chronic kidney disease; eGFR, estimated glomerular filtration rate; BUN, blood urea nitrogen; UA, uric acid; CysC, cystatin C. Bold values indicate statistical significance (*P* < 0.05).

### 3.2 Preoperative baseline RI and PTRI prevalence in patients with CRC

**FIGURE 2:**
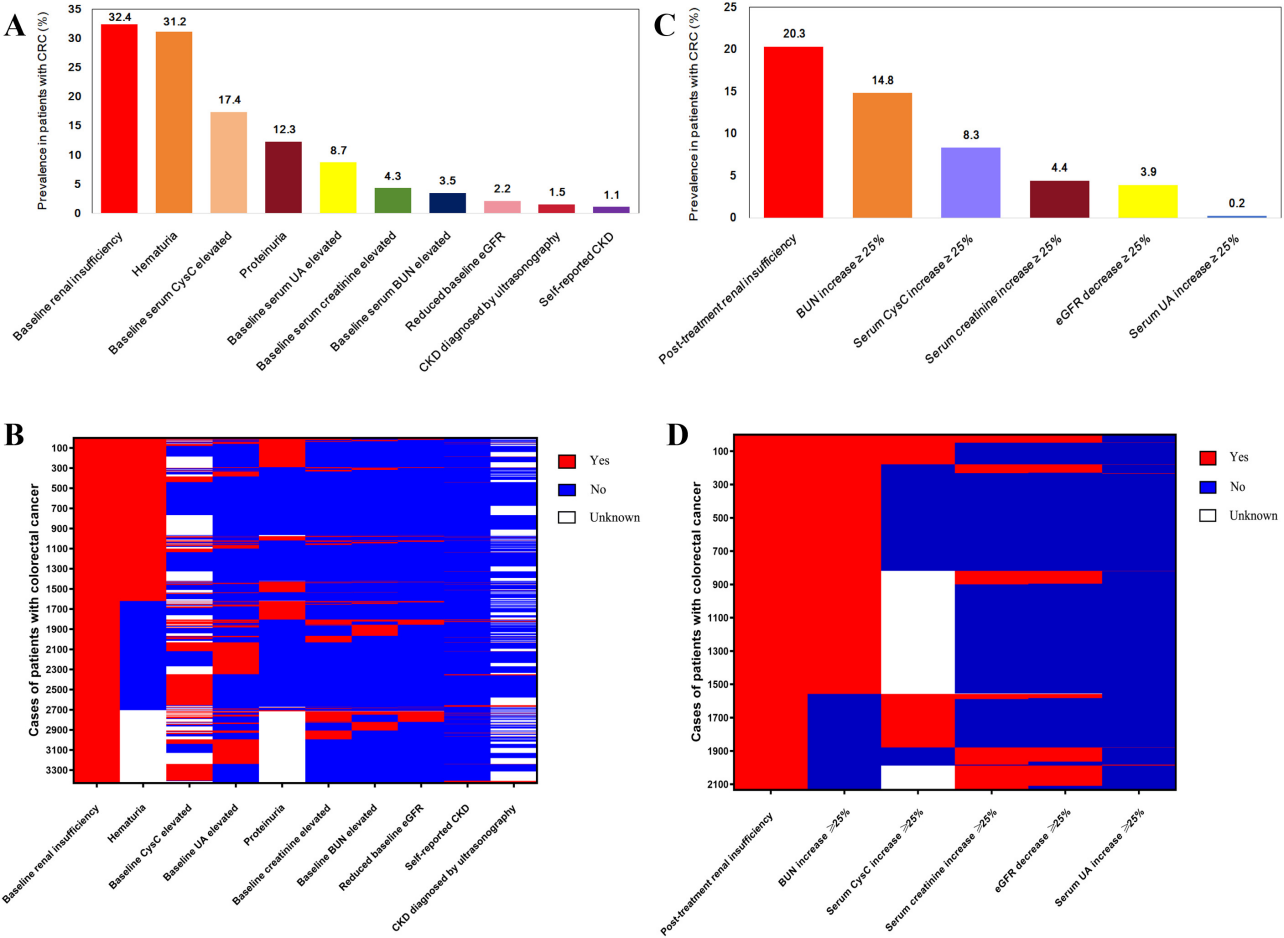
Prevalence and distribution of preoperative baseline renal insufficiency **(A,B)** and post-treatment renal insufficiency **(C,D)** related parameters in patients with colorectal cancer. (CKD, chronic kidney disease; eGFR, estimated glomerular filtration rate; Reduced baseline eGFR was defined as baseline eGFR < 60 mL/min/1.73 m^2^; BUN, blood urea nitrogen; UA, uric acid; CysC, cystatin C).

Among the 10,581 patients, 3,427 (32.4%) had preoperative baseline RI. Prevalence of baseline RI-related parameters is: hematuria (1,618/5,194; 31.2%), elevated baseline serum CysC (1,050/6,029; 17.4%), proteinuria (639/5,195; 12.3%), elevated baseline serum UA (917/10,488; 8.7%), elevated baseline serum creatinine (456/10,484; 4.3%), elevated baseline BUN (370/10,487; 3.5%), reduced baseline eGFR (227/10,484; 2.2%), CKD diagnosed by ultrasonography (99/6,460; 1.5%) and self-reported CKD (117/10,581; 1.1%) (Figure 2A; Supplementary Tables 1, 2). The distribution of baseline RI-related parameters in the 3,427 patients with CRC is shown in Figure 2B. From Figure 2B, we could observe that about half of RI (1,618/3,427; 47.2%) was attributed to hematuria, with other RI-related parameters providing complementary insights.

We have performed a sensitivity analysis comparing our original “comprehensive” definition of baseline RI with a stricter definition based solely on KDIGO 2024 criteria (eGFR < 60 mL/min/1.73 m^2^ and/or documented albuminuria/proteinuria) (Supplementary Table 3). As shown in the newly added (Supplementary Table 3), compared with baseline RI, the sensitivities of baseline eGFR, proteinuria and hematuria are 6.6, 23.7 and 59.9%, respectively. The comprehensive definition of RI demonstrated a significant increase in the sensitivity of detecting renal impairment. These results suggest that incorporating additional clinical indicators beyond strict KDIGO criteria (e.g., reduced eGFR, proteinuria, or hematuria) may capture a broader at-risk population and enhance prognostic stratification.

We have compared baseline characteristics between patients who did (*N* = 5,194) and did not (*N* = 5,387) receive urinalysis (Supplementary Table 4). The comparison includes age, sex, TNM stage, eGFR, hypertension, diabetes, cardiovascular disease, and other relevant comorbidities. Preliminary analysis indicates that patients who received urinalysis tend to have more CKD (1.5% vs. 0.7%, *p* < 0.001), more urinary disease (10.5% vs. 6.3%, *p* < 0.001), more respiratory disease (1.4% vs. 1.0%, *p* = 0.024) and less hypertension (30.2% vs. 32.3%, *p* = 0.020) (Supplementary Table 4), compared to those without urinalysis. No significant difference was found in terms of sex, age, baseline eGFR and TNM stage (Supplementary Table 4). Regarding peak post-treatment serum levels, 1,272 (21.1%) patients exhibited elevated serum levels of CysC, 938 (8.9%) had elevated UA, 743 (7.1%) had elevated creatinine, and 741 (7.1%) had elevated BUN (Supplementary Table 2). Among the patients, only 387 (3.7%) exhibited reduced eGFR based on the lowest eGFR (Supplementary Tables 1, 2).

Among the 2,132 (20.3%) patients with PTRI, 1,557 (14.8%) had a ≥ 25% increase in BUN, 500 (8.3%) had a ≥ 25% increase in serum CysC, 458 (4.4%) had a ≥ 25% increase in serum creatinine, 405 (3.9%) experienced a ≥ 25% decrease in eGFR, and 17 (0.2%) had a ≥ 25% increase in serum UA (Figures 2C,D; Supplementary Table 5). From Figure 2D, it is evident that most PTRI (73.0%; 1,557/2,132) was attributed to BUN increase, with other PTRI-related parameters complementing this finding.

### 3.3 Relationship between preoperative baseline RI and clinicopathological parameters in patients with CRC

**FIGURE 3:**
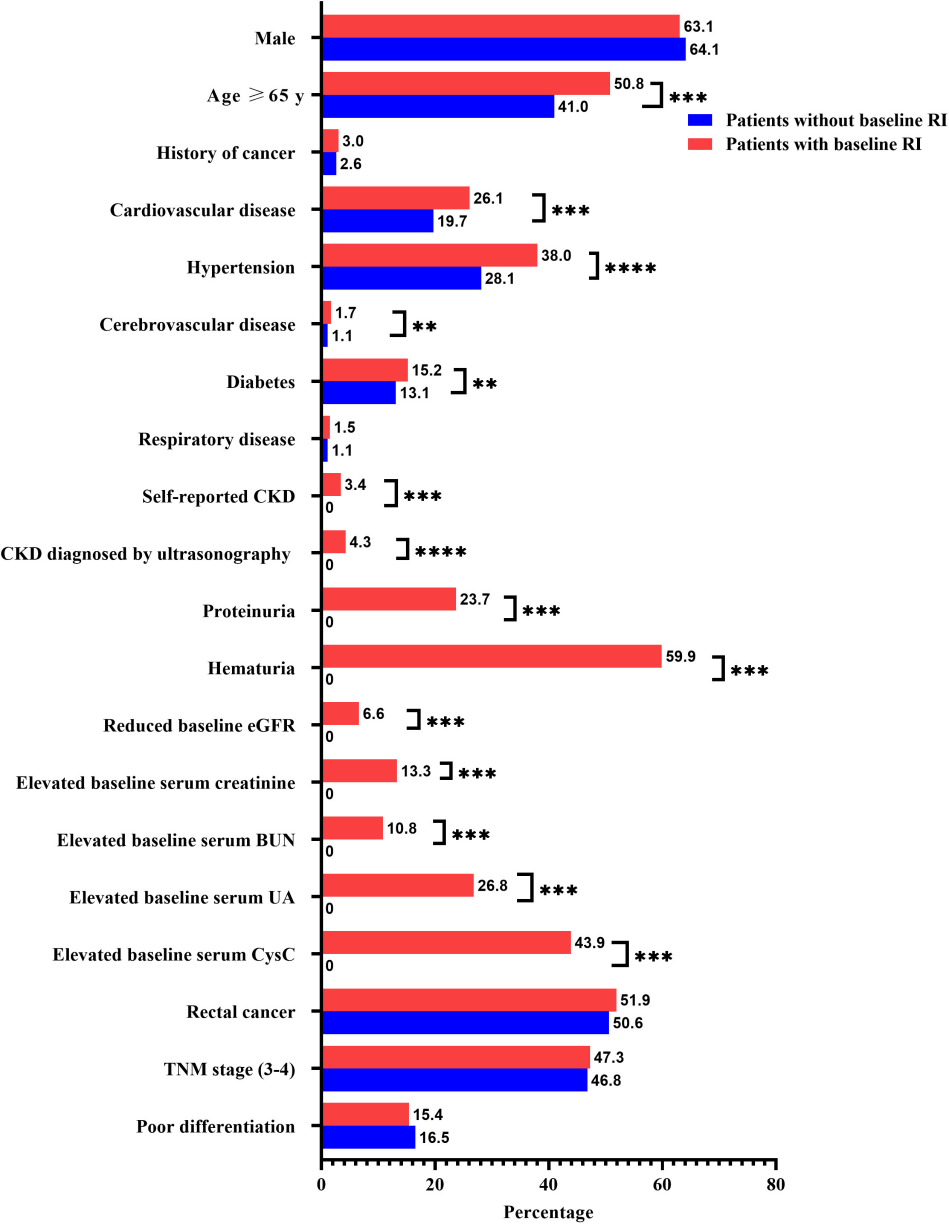
Relationship between preoperative baseline renal insufficiency (RI) and categorical parameters in patients with colorectal cancer. BMI, body mass index; CKD, chronic kidney disease; eGFR, estimated glomerular filtration rate; Reduced baseline eGFR was defined as baseline eGFR < 60 mL/min/1.73 m^2^; BUN, blood urea nitrogen; UA, uric acid; CysC, cystatin C; **P < 0.01; ***P < 0.001.

Compared with patients without baseline RI, patients with baseline RI had significantly older age, more history of cardiovascular disease, hypertension, cerebrovascular disease, and diabetes. In addition, they had lower levels of WBC and neutrophils, higher levels of lymphocytes, and prolonged hospital stay (all *P* < 0.05) (Tables 1, 2; Figure 3).

### 3.4 Univariate and multivariate logistic regression analysis of preoperative baseline RI risk factors in CRC patients

Univariate logistic regression identified age ≥ 65 years, BMI ≥ 24 kg/m^2^, history of cardiovascular disease, hypertension, cerebrovascular disease, and diabetes as significant risk factors for baseline RI (all *P* < 0.05; Table 3). Multivariate analysis further revealed that age ≥ 65 years [Hazard ratio (HR): 1.298, 95% CI: 1.163–1.449, *P* < 0.001] and history of cardiovascular disease (HR: 1.513, 95% CI: 1.353–1.693, *P* < 0.001) remained independent predictors of baseline RI (Table 3).

**TABLE 3:** Univariate and multivariate logistics analysis of risk factors for preoperative baseline renal insufficiency in patients with colorectal cancer.

Parameters	Univariate analysis		Multivariate analysis	
	HR (95%CI)	P*	HR (95%CI)	*P* *
Age (≥ 65 vs. < 65 year)	1.486 (1.369–1.613)	** < 0.001**	1.298 (1.163–1.449)	** < 0.001**
BMI (≥ 24 vs. < 24 kg/m^2^)*	1.151 (1.034–1.280)	**0.010**	1.089 (0.976–1.215)	0.126
Cardiovascular disease (yes vs. no)	1.436 (1.304–1.580)	** < 0.001**	1.513 (1.353–1.693)	** < 0.001**
Hypertension (yes vs. no)	1.571 (1.442–1.713)	** < 0.001**	1.115 (0.909–1.368)	0.297
Cerebrovascular disease (yes vs. no)	1.631 (1.158–2.298)	**0.005**	1.329 (0.937–1.886)	0.111
Diabetes (yes vs. no)	1.188 (1.058–1.334)	**0.004**	0.948 (0.809–1.111)	0.507

*BMI, body mass index. Bold values indicate statistical significance (*P* < 0.05).

### 3.5 Univariate and multivariate logistic regression analysis of PTRI risk factors in patients with CRC

Comparing patients with and without PTRI, those with PTRI showed higher rates of open surgery, postoperative complications, intestinal ostomy, blood transfusion, and postoperative chemoradiotherapy (all *P* < 0.05) (Supplementary Figure 1; Supplementary Table 6).

Univariate logistic regression analysis revealed that open surgery, postoperative complications, intestinal ostomy, blood transfusion, and postoperative chemoradiotherapy were risk factors for PTRI (Supplementary Table 7). Multivariate analysis demonstrated that open surgery (HR: 1.252, 95% CI: 1.089–1.440, *P* = 0.002), intestinal ostomy (HR: 1.235, 95% CI: 1.073–1.421, *P* = 0.003), postoperative complications (HR: 2.398, 95% CI: 1.653–3.477, *P* < 0.001) and postoperative chemoradiotherapy (HR: 2.071, 95% CI: 1.413–3.486, *P* < 0.001) were independent risk factors for PTRI (Supplementary Table 7).

### 3.6 Kaplan-Meier analysis of prognostic factors for DFS and OS in CRC patients

**FIGURE 4:**
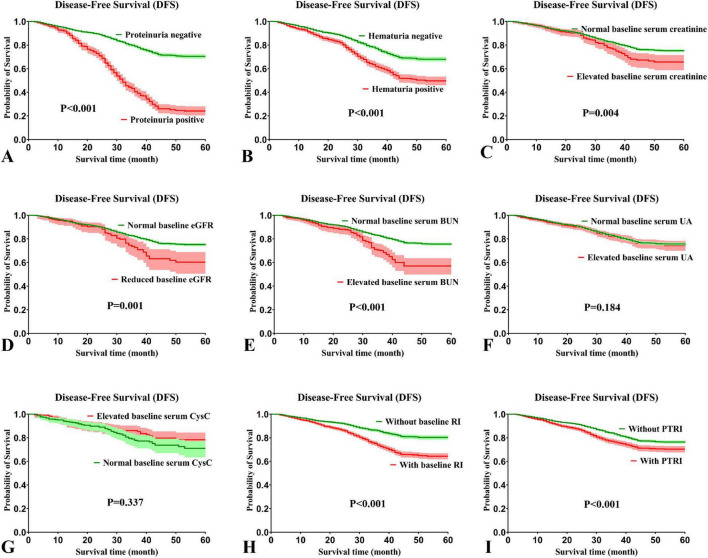
Effects of various kidney function-related parameters on disease-free survival in patients with colorectal cancer. **(A)** Proteinuria (*P* < 0.001); **(B)** hematuria (*P* < 0.001); **(C)** baseline serum level of creatinine (*P* = 0.004); **(D)** baseline estimated glomerular filtration rate (eGFR, *P* = 0.001); **(E)** baseline serum level of blood urea nitrogen (BUN, *P* < 0.001); **(F)** baseline serum level of uric acid (UA, *P* = 0.184); **(G)** baseline serum level of cystatin C (CysC, *P* = 0.337); **(H)** preoperative baseline renal insufficiency (RI, *P* < 0.001); **(I)** post-treatment renal insufficiency (PTRI, *P* < 0.001).

Kaplan-Meier analysis showed that proteinuria, hematuria, elevated baseline serum creatinine, lower baseline eGFR, elevated baseline serum BUN, baseline RI and PTRI were associated with shorter DFS (all *P* < 0.05) (Figure 4). In contrast, no significant correlations were observed between baseline serum UA, baseline serum CysC and DFS.

Similarly, proteinuria, hematuria, lower baseline eGFR, elevated baseline serum BUN, baseline RI and PTRI were associated with shorter OS (all *P* < 0.05) (Supplementary Figure 2). However, no significant relationship was noted between baseline serum levels of creatinine, UA, CysC, and OS.

### 3.7 Univariate and multivariate COX regression analysis of prognostic factors for DFS and OS in CRC patients

The univariate and multivariate Cox proportional hazards regression analysis demonstrated that both baseline RI (HR: 1.885, 95% CI: 1.632–2.179, *P* < 0.001), and PTRI (HR: 1.337, 95% CI: 1.134–1.578, *P* = 0.001) were independent prognostic factors for adverse DFS (Supplementary Table 8; Table 4). Similarly, baseline RI (HR: 1.721, 95%CI: 1.468–2.016, *P* < 0.001), and PTRI (HR: 1.345, 95% CI: 1.123–1.610, *P* = 0.001) were independent prognostic factors for adverse OS (Supplementary Table 8; Table 4).

**TABLE 4:** Multivariate COX analysis of risk factors for disease-free survival and overall survival in patients with colorectal cancer.

Parameters	Disease-free survival		Overall survival	
	HR (95%CI)	P*	HR (95%CI)	*P* *
Preoperative baseline renal insufficiency (RI, yes vs. no)	1.885 (1.632–2.179)	**<0.001**	1.721 (1.468–2.016)	**<0.001**
Post-treatment renal insufficiency (PTRI, yes vs. no)	1.337 (1.134–1.578)	**0.001**	1.345 (1.123–1.610)	**0.001**
Radical resection (palliative vs. radical)	1.805 (1.397–2.332)	**<0.001**	1.611 (1.202–2.160)	**0.001**
TNM stage (III-IV vs. I-II)	1.234 (1.062–1.433)	**0.006**	1.332 (1.130–1.571)	**0.001**
Vascular invasion (positive vs. negative)	2.525 (2.163–2.949)	**<0.001**	2.341 (1.975–2.774)	**<0.001**
Perineural invasion (positive vs. negative)	1.238 (1.059–1.448)	**0.008**	1.302 (1.098–1.544)	**0.002**
Tumor deposits (positive vs. negative)	1.244 (1.055–1.467)	**0.009**	1.227 (1.025–1.469)	**0.026**
Tumor invasion of adjacent organs (T4b, yes vs. no)	1.234 (0.876–1.723)	0.182	1.251 (0.732–1.872)	0.236

*Bold values indicate statistical significance (*P* < 0.05).

Sensitivity analysis had been performed by excluding patients with pre-existing CKD (defined as baseline eGFR < 60 mL/min/1.73 m^2^ or documented CKD diagnosis in medical records). This restricted cohort included 10,268 patients, and the results remained consistent with our primary findings: the adjusted prevalence of baseline RI (30.3%) and PTRI (20.0%) were still very high, and both baseline RI and PTRI were still independent predictors of DFS and OS (Supplementary Table 9).

## 4 Discussion

This retrospective cohort study analyzed 10,581 CRC patients treated at Changhai Hospital over a 5-year period to investigate the prevalence, risk factors and clinical impact of baseline RI and PTRI. The study revealed a preoperative baseline RI prevalence of 32.4%, with hematuria accounting for 47.2% of cases. Other RI-related parameters provided complementary diagnostic value to hematuria. Multivariate analysis identified age ≥ 65 years and preexisting cardiovascular disease as independent risk factors for baseline RI. Post-treatment evaluation showed 2,132 patients (20.3%) developed PTRI, primarily driven by BUN elevation (73.0% of cases), with other PTRI-related parameters offering Supplementary diagnostic information. Independent risk factors for PTRI included open surgery, intestinal ostomy, postoperative complications, and postoperative chemoradiotherapy. Both baseline RI and PTRI independently predicted worse DFS and OS. These findings demonstrate the significant clinical burden of RI in CRC patients throughout the treatment continuum, with distinct risk factor profiles for baseline RI versus PTRI.

To the best of our knowledge, this represents the first comprehensive study systematically evaluating the prevalence, risk factors, and prognostic significance of baseline RI, PTRI, and associated parameters in CRC patients. We propose the novel concept of “baseline RI” as an alternative to the conventional CKD classification. According to the KDIGO 2012 Clinical Practice Guideline for the Evaluation and Management of Chronic Kidney Disease (18), CKD was defined as abnormalities of kidney structure or function lasting for > 3 months (18), including GFR < 60 mL/min/1.73 m^2^ or any one of the following criteria: ➀ albuminuria [albumin excretion rate (AER) ≥ 30 mg/24 h; ACR ≥ 30 mg/g]; ➁ urine sediment abnormalities; ➂ electrolyte and other abnormalities due to tubular disorders; ➃ abnormalities detected by histology; ➄ structural abnormalities detected by imaging; ➅ history of kidney transplantation (18). However, this CKD definition presents notable limitations in CRC research: First, retrospective studies often lack sufficient data to accurately assess the 3-month duration requirement. Second, routine CRC clinical practice typically does not include AER or ACR measurements in standard urinalysis. Consequently, prior CRC studies have variably defined CKD based on reduced eGFR (10, 12, 13, 24, 25), albuminuria, proteinuria, or hematuria (3, 13, 20, 21). Our “baseline RI” concept provides a clinically practical alternative, incorporating: (1) CKD history, (2) reduced eGFR, and (3) abnormalities detected via urinalysis, blood tests, or ultrasonography.

Previous studies with small sample size reported that the prevalence of reduced eGFR in CRC patients ranged from 9.6 to 26.8% (10, 12, 13, 24, 25). In studies where CKD definition is ambiguous, prevalence of CKD in patients with CRC ranged from 2.4 to 15.8% (4, 11, 26, 27). Proteinuria occurs more frequently in CRC patients than in the general population. Our prevalence of proteinuria (12.3%) in CRC patients is also substantially higher than the corresponding prevalence of 6.7% in the general population (3). A 1998–2009 cohort study of 3,379 CRC patients found that 14.6% exhibited proteinuria (14), consistent with our findings. Similarly, a 2009 Polish population study using turbidimetric methods reported an 11.9% proteinuria prevalence (28). Among 4,684 solid tumor patients, 7.2% had elevated serum creatinine, while only 4.1% showed reduced eGFR (29), highlighting discrepancies between these renal markers. In our study, RI prevalence reached 32.4% in CRC patients, yet reduced eGFR was observed in only 2.2%—a rate comparable to the general Chinese population (3). This suggests that eGFR alone fails to capture the full spectrum of kidney dysfunction in CRC patients. Collectively, our proposed baseline RI and PTRI metrics offer a more clinically practical and comprehensive approach to assessing renal impairment than the traditional CKD or eGFR criteria.

Established risk factors for CKD include older age, female sex, smoking history, obesity, hypertension, diabetes, dyslipidemia, and cardiovascular disease (3, 4, 9). Our study corroborates these findings, identifying older age, obesity, hypertension, diabetes, cardiovascular disease, and cerebrovascular disease as significant risk factors for baseline RI. In our cohort, the prevalence of cardiovascular disease, hypertension, and diabetes was 21.8, 31.3, and 13.8%, respectively—substantially lower than the rates reported by Teng et al. (55.2, 57.4, and 20.9%) (11). Similarly, Kozłowski et al. observed higher prevalences of hypertension (62%) and diabetes (23%) in CRC patients, potentially attributable to their older study population (4). Notably, while advanced TNM stage has been linked to increased proteinuria incidence (14), we found no significant association between baseline RI and TNM stage.

A Danish cohort study of 6,580 CRC surgical patients (2005–2011) defined acute kidney injury (AKI) as either: (1) ≥ 50% increase in plasma creatinine within 7 postoperative days, or (2) absolute creatinine rise ≥ 26 μmol/L within 48 h (17). This study reported AKI incidence of 20.3% (1,337/6,580), correlating with increased 90-day mortality (17). Notably, this AKI rate matches our observed PTRI incidence (20.3%). Consistent with prior reports (9), we confirmed associations between PTRI and both blood transfusions and perioperative complications. Chemoradiotherapy specifically elevates serum creatinine, induces proteinuria, and increases PTRI risk (13, 30, 31). For example, Rödel et al. documented creatinine elevation (18%) and proteinuria (16%) in 104 preoperative chemotherapy recipients (30). Common agents (5-fluorouracil, capecitabine, oxaliplatin) induce tubular injury (13), while radiotherapy causes DNA oxidative damage contributing to PTRI (13). Clinically, PTRI represents the second-leading cause of postoperative chemotherapy ineligibility in CRC (27) and exacerbates chemotherapy cardiotoxicity (32, 33), pulmonary infections, cardiovascular complications, and 30-day mortality (25, 34). Baseline RI independently predicts mortality in general populations (35), cardiovascular risk and overall mortality in cancer patients (21), worse OS and DFS in CRC patients (4, 9, 12, 14, 25, 34). These findings align with our results. In addition, PTRI was reported to be associated with increased 90-day mortality (17), which is consistent with our findings.

As noted, our definition of PTRI, based on a ≥ 25% increase in BUN, creatinine, UA, or CysC from baseline level—yielded a relatively high incidence (20.3%). We acknowledge that this metric likely captures hemodynamic stress or systemic catabolic states rather than true AKI. PTRI defined in this study is not equivalent to AKI and should be interpreted as a surrogate for perioperative physiological stress. Creatinine is a more reliable marker of GFR, as it is primarily eliminated by renal filtration with minimal tubular reabsorption. BUN, in contrast, is highly sensitive to non-renal factors, including dehydration, gastrointestinal (GI) bleeding, high-protein catabolism, corticosteroid use, and activation of neurohormonal systems such as the rennin-angiotensin-aldosterone system (RAAS) and sympathetic nervous system (36–39). GI bleeding is a specific confounder in colorectal surgery. Postoperative GI bleeding—a known complication after colorectal resection—can markedly elevate BUN through increased protein load from digested blood, without any change in GFR. This is particularly relevant in our cohort and may partially explain the high PTRI incidence. To directly contrast “hemodynamic stress” versus “true renal injury,” we have also calculated the incidence of postoperative AKI using the KDIGO creatinine criterion: an absolute increase in serum creatinine of ≥ 0.3 mg/dL within 48 h postoperatively. The incidence of KDIGO-defined AKI (≥ 0.3 mg/dL increase in serum creatinine) was 2.2%, substantially lower than the 20.3% PTRI rate based on BUN, creatinine, UA, and CysC. Therefore, the definition of PTRI in our study is not equal to the definition of AKI in 2012 KDIGO guideline.

This study proposes two novel clinical concepts: baseline RI and PTRI, providing a comprehensive framework for assessing kidney dysfunction in CRC patients. These classifications offer superior clinical utility by encompassing the complete spectrum of renal injury, with significant implications for both research and clinical practice. Our prospective cohort study (*n* = 10,581) revealed: (1) high prevalence rates of RI in CRC patients (baseline RI: 32.4%; PTRI: 20.3%); (2) risk factors of RI [older age (HR = 1.298), and cardiovascular disease (HR = 1.513)]; (3) risk factors of PTRI [open surgery (HR = 1.252), intestinal ostomy (HR = 1.235), postoperative complications (HR = 2.398), and postoperative chemoradiotherapy (HR = 2.071)]; (4) significant associations with reduced DFS and OS. These findings necessitate: (1) protocolized renal monitoring for high-risk patients (age ≥ 65 years, concomitant cardiovascular disease) (40, 41); (2) preventive strategies including optimized hydration regimens, especially for patients with open surgery, intestinal ostomy, postoperative complications and chemoradiotherapy; (3) adaptive chemotherapy dosing algorithms (42). Chemotherapy-induced PTRI necessitated dose reduction and treatment discontinuation, significantly compromising oncological outcomes (13). Since hypertension, diabetes, and cardiovascular diseases are common concomitant diseases, and they are associated with higher prevalence of baseline RI, stringent control of hypertension and diabetes is required to mitigate renal toxicity. While the pathophysiology requires further elucidation (4), emerging evidence implicates paraneoplastic mechanisms, particularly tumor-derived cytokines triggering renal immunological and inflammatory cascade reactions (14, 43).

Several study limitations should be considered when interpreting our findings. First, some non-surgical CRC cases were excluded, such as those treated with endoscopic resection for early CRC, and low rectal cancer cases with clinical complete response after preoperative chemoradiotherapy. Second, urinalysis was performed in approximately 50% of participants, potentially introducing selection bias in renal function assessment. Patients who received urinalysis were more likely to have CKD (1.5% vs. 0.7%, *p* < 0.001) and urinary disease (10.5% vs. 6.3%, *p* < 0.001), compared to those without urinalysis. These findings suggest the presence of indication bias: clinicians were more likely to order urinalysis in patients with urinary disease and CKD, which may have led to an overestimation of hematuria and proteinuria prevalence in our cohort. Third, single-time point urinalysis limited our ability to assess treatment-related changes in proteinuria/hematuria dynamics. Fourth, our operational definition of proteinuria diverged from the KDIGO standard (proteinuria is usually defined as urine ACR of > 30 mg/g or AER ≥ 30 mg/24 h) (3, 44). This adaptation was necessary because 24-h urine collections are not standard in CRC management protocols. Our pragmatic approach utilized routinely available dipstick measurements, ensuring feasibility while maintaining diagnostic validity (20, 21). Fifth, while our sample provides valuable insights into CRC management and outcomes within a high-volume academic center—a common setting for complex oncology care in China—it may not fully capture the heterogeneity of CRC across diverse geographic regions, healthcare tiers, or rural–urban divides in the country. Future multicenter, population-based studies involving hospitals at various levels and regions would be essential to validate our findings and enhance external validity. Nonetheless, given the central role that tertiary hospitals play in CRC diagnosis and treatment nationwide, our results still offer clinically relevant information that may inform practice and policy, particularly in similar referral centers.

## 5 Conclusion

This retrospective cohort study demonstrates a substantial burden of renal impairment in CRC patients, with baseline RI and PTRI prevalence rates of 32.4 and 20.3%, respectively. The composite baseline RI and PTRI assessment [incorporating eGFR, urinalysis (proteinuria/hematuria), serum biomarkers (creatinine, BUN, uric acid, CysC), and CKD history], provides more comprehensive renal evaluation than isolated eGFR or CKD classification. Univariate and multivariate analysis demonstrated that older age and cardiovascular disease were independent risk factors for baseline RI; while open surgery, intestinal ostomy, postoperative complications and chemoradiotherapy were independent risk factors for PTRI. Both baseline RI and PTRI were independent risk factors for adverse DFS and OS. We recommend comprehensive management for high-risk patients: (1) preoperative: renal optimization protocols (hydration, comorbidity control); (2) intraoperative: nephroprotective strategies such as goal-directed fluid therapy, and choose laparoscopic surgery instead of open surgery; (3) postoperative: enhanced monitoring schedules especially for patients with intestinal ostomy and postoperative complications, and adaptive chemoradiotherapy dosing algorithms. To validate our findings, further multicenter prospective studies with larger sample sizes are warranted.

## Author’s note

This study had been presented as a poster at the 5th International Congress of Chinese Nephrologists cum Hong Kong Society of Nephrology Annual Scientific Meeting 2024 (ICCN 2024) which was held on 13–15 December 2024, at the Hong Kong. It had also been presented as a poster at the Congress of the Chinese Society of Nephrology 2024 (CCSN2024) which was held on 16-20 October 2024 at Chongqing, China. It was also accepted as an oral presentation at the 2025 CSN Critical Care & Blood Purification Congress (CCBPC 2025) which will be held on 2–5 July 2025 at Tianjin, China.

## Data Availability

The original contributions presented in this study are included in this article/Supplementary material, further inquiries can be directed to the corresponding authors.
