# Supplementary material for: Prevalence and prognostic value of baseline and post-treatment renal insufficiency in colorectal cancer patients: a large retrospective cohort study of 10,581 patients in Shanghai, China (2019–2023)
Source: Front Med (Lausanne). 2026 Jan 13;12:1671990. doi: 10.3389/fmed.2025.1671990 (PMC12836388; doi:10.3389/fmed.2025.1671990)
Supplement: Supplementary file 1 [file Data_Sheet_1.docx]

**Supplementary tables**

**
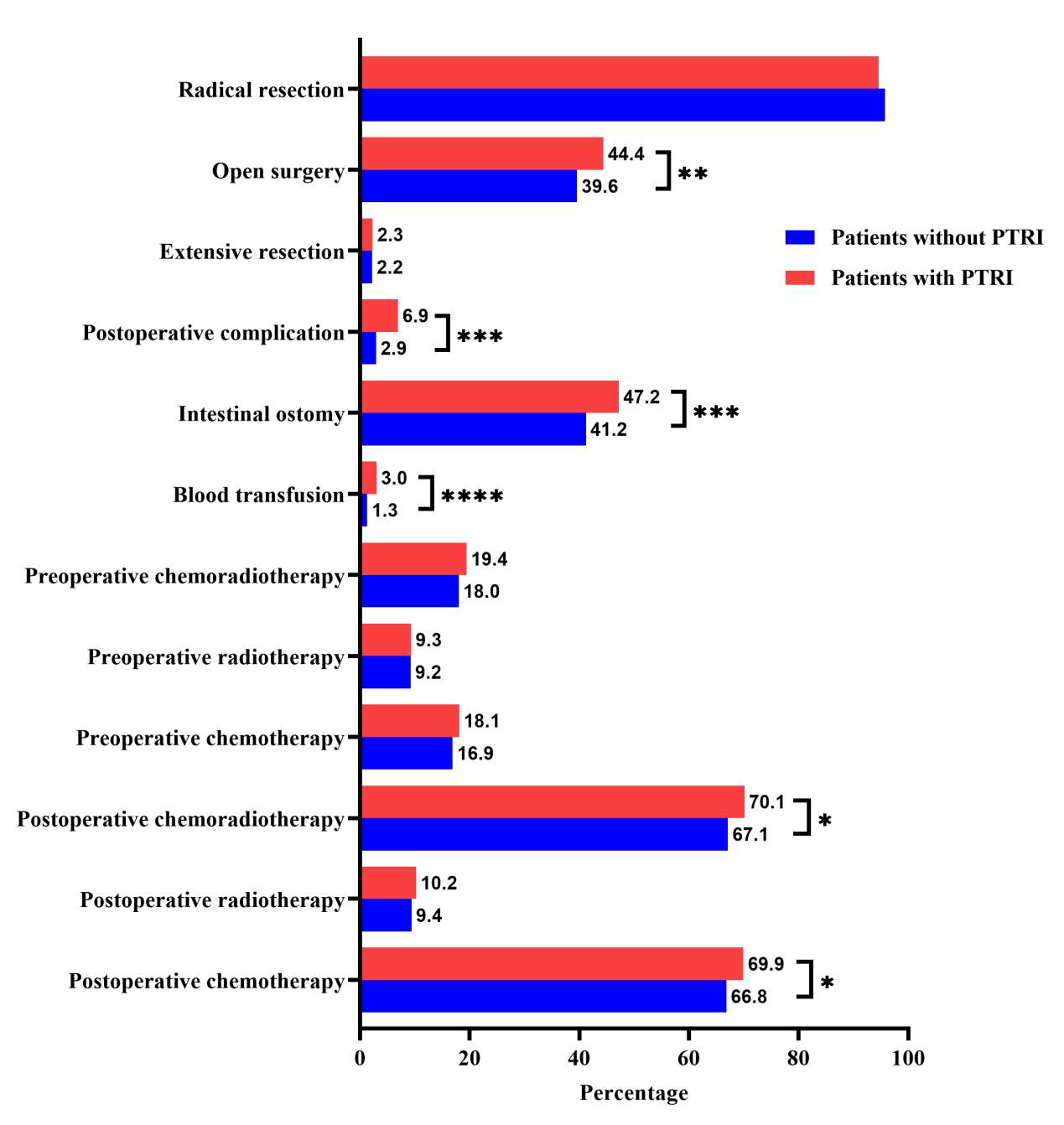
**

**Fig S1.** Relationship between post-treatment renal insufficiency (PTRI) and categorical parameters in patients with colorectal cancer (**P* < 0.05, ***P* < 0.01, ****P* < 0.001)


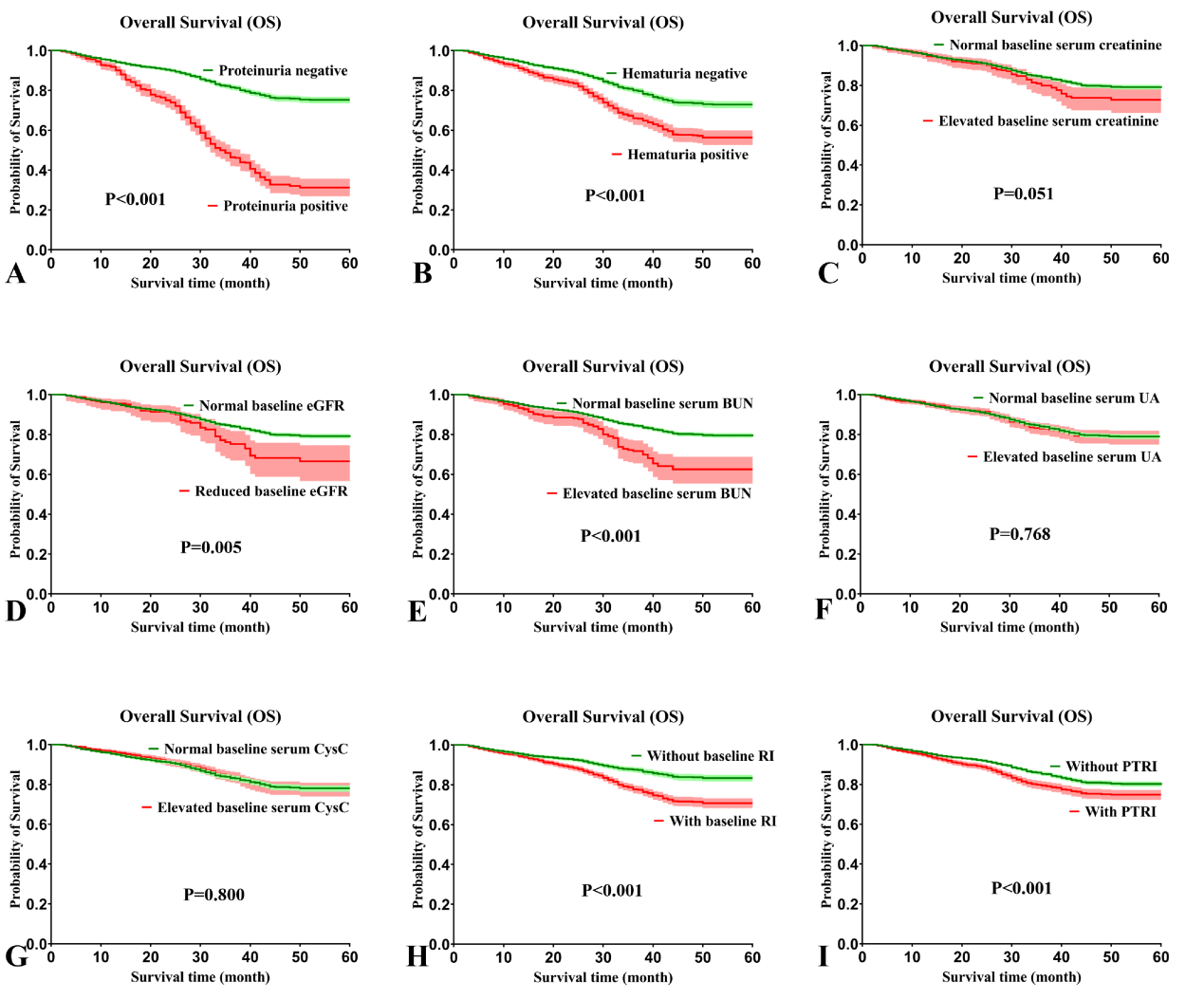


**Fig S2**. Effects of various kidney function-related parameters on overall survival in patients with colorectal cancer. A: proteinuria (***P* < 0.001**); B: hematuria (***P* < 0.001**); C: baseline serum level of creatinine (*P* = 0.051); D: baseline estimated glomerular filtration rate (eGFR, ***P* = 0.005**); E: baseline serum level of blood urea nitrogen (BUN, ***P* < 0.001**); F: baseline serum level of uric acid (UA, *P* = 0.768); G: baseline serum level of cystatin C (CysC, *P* = 0.800); H: preoperative baseline renal insufficiency (RI, ***P* < 0.001**); I: post-treatment renal insufficiency (PTRI, ***P* < 0.001**).

**Table S1.**The distribution of estimated glomerular filtration rate (eGFR) in patients with colorectal cancer

| Timing of eGFR evaluation | Stage of CKD* | eGFR | Cases | Percentage |
| --- | --- | --- | --- | --- |
| The baseline eGFR at the diagnosis of colorectal cancer (mL/min/1.73m^2^) | 1 | ≥90 | 8145 | 77.7% |
|  | 2 | 60-89 | 2112 | 20.1% |
|  | 3 | 30-59 | 213 | 2.0% |
|  | 4 | 15-29 | 13 | 0.1% |
|  | 5 | <15 | 1 | 0.0% |
|  |  | Unknown | 97 |  |
|  | Total |  | 10581 |  |
| The lowest eGFR after the treatment of colorectal cancer (mL/min/1.73m^2^) | 1 | ≥90 | 7337 | 70.2% |
|  | 2 | 60-89 | 2734 | 26.1% |
|  | 3 | 30-59 | 340 | 3.3% |
|  | 4 | 15-29 | 45 | 0.4% |
|  | 5 | <15 | 2 | 0.0% |
|  |  | Unknown | 123 |  |
|  | Total |  | 10581 |  |

*CKD, chronic kidney disease.

**Table S2.**The prevalence of preoperative baseline renal insufficiency (RI) and related parameter in patients with colorectal cancer

| Parameters | Prevalence | Yes | No | Unknown |
| --- | --- | --- | --- | --- |
| Self-reported CKD | 1.1% | 117 | 10464 | 0 |
| CKD diagnosed by ultrasonography | 1.5% | 99 | 6361 | 4121 |
| Proteinuria | 12.3% | 639 | 4556 | 5386 |
| Hematuria | 31.2% | 1618 | 3576 | 5387 |
| Baseline eGFR < 60 mL/min/1.73m^2^ | 2.2% | 227 | 10257 | 97 |
| Lowest eGFR < 60 mL/min/1.73m^2^ | 3.7% | 387 | 10071 | 123 |
| Baseline serum creatinine elevated | 4.3% | 456 | 10028 | 97 |
| Peak serum creatinine elevated | 7.1% | 743 | 9715 | 123 |
| Baseline serum BUN elevated | 3.5% | 370 | 10117 | 94 |
| Peak serum BUN elevated | 7.1% | 741 | 9746 | 94 |
| Baseline serum UA elevated | 8.7% | 917 | 9571 | 93 |
| Peak serum UA elevated | 8.9% | 938 | 9550 | 93 |
| Baseline serum CysC elevated | 17.4% | 1050 | 4979 | 4552 |
| Peak serum CysC elevated | 21.1% | 1272 | 4757 | 4552 |
| Preoperative baseline renal insufficiency (RI) | 32.4% | 3427 | 7154 | 0 |

*CKD, chronic kidney disease; eGFR, estimated glomerular filtration rate; BUN, blood urea nitrogen; UA, uric acid; CysC, cystatin C.

**Table S3**. The sensitivity of various kidney impairment related parameters compared with renal insufficiency (RI).

| Kidney impairment related parameters |  | Renal insufficiency | | Sensitivity |
| --- | --- | --- | --- | --- |
|  |  | No | Yes |  |
| Self-reported CKD |  |  |  | 3.4% |
|  | No | 7154 | 3310 |  |
|  | Yes | 0 | 117 |  |
| CKD diagnosed by ultrasonography |  |  |  | 4.3% |
|  | No | 4156 | 2205 |  |
|  | Yes | 0 | 99 |  |
| Proteinuria |  |  |  | 23.7% |
|  | Negative | 2503 | 2053 |  |
|  | Positive | 0 | 639 |  |
| Hematuria |  |  |  | 59.9% |
|  | Negative | 2489 | 1086 |  |
|  | Positive | 0 | 1619 |  |
| Baseline eGFR |  |  |  | 6.6% |
|  | >=60 mL/min/1.73 m² | 7063 | 3194 |  |
|  | <60 mL/min/1.73 m² | 0 | 227 |  |
| Baseline serum creatinine |  |  |  | 13.3% |
|  | Normal | 7063 | 2965 |  |
|  | Elevated | 0 | 456 |  |
| Baseline serum BUN |  |  |  | 10.8% |
|  | Normal | 7066 | 3051 |  |
|  | Elevated | 0 | 370 |  |
| Baseline serum UA |  |  |  | 26.8% |
|  | Normal | 7067 | 2504 |  |
|  | Elevated | 0 | 917 |  |
| Baseline serum CysC |  |  |  | 43.9% |
|  | Normal | 3636 | 1343 |  |
|  | Elevated | 0 | 1050 |  |

**Table S4.** Baseline characteristics between patients who did and did not receive urinalysis.

| Parameters |  | Patients without urinalysis  (N=5387) | Patients with urinalysis  (N=519‍4) | Total  (N=10581) | *P* |
| --- | --- | --- | --- | --- | --- |
| Sex | Male | 3432(63.7%) | 3315(63.8%) | 6747(63.8%) | 0.902 |
|  | Female | 1955(36.3%) | 1879(36.2%) | 3834(36.2%) |  |
| Age | <65y | 2985(55.4%) | 2919(56.2%) | 5904(55.8%) | 0.414 |
|  | >=65y | 2402(44.6%) | 2275(43.8%) | 4677(44.2%) |  |
| Hypertension | No | 3647(67.7%) | 3625(69.8%) | 7272(68.7%) | **0.020** |
|  | Yes | 1740(32.3%) | 1569(30.2%) | 3309(31.3%) |  |
| Diabetes | No | 4655(86.4%) | 4467(86.0%) | 9122(86.2%) | 0.542 |
|  | Yes | 732(13.6%) | 727(14.0%) | 1459(13.8%) |  |
| Respiratory disease | No | 5335(99.0%) | 5119(98.6%) | 10454(98.8%) | **0.024** |
|  | Yes | 52(1.0%) | 75(1.4%) | 127(1.2%) |  |
| Urinary disease | No | 5049(93.7%) | 4649(89.5%) | 9698(91.7%) | **<0.001** |
|  | Yes | 338(6.3%) | 545(10.5%) | 883(8.3%) |  |
| CKD | No | 5350(99.3%) | 5114(98.5%) | 10464(98.9%) | **<0.001** |
|  | Yes | 37(0.7%) | 80(1.5%) | 117(1.1%) |  |
| Baseline eGFR | >=60 mL/min/1.73 m² | 5188(98.0%) | 5069(97.7%) | 10257(97.8%) | 0.305 |
|  | <60 mL/min/1.73 m² | 107(2.0%) | 120(2.3%) | 227(2.2%) |  |
| Postoperative TNM stage | I-II | 2622(53.0%) | 2515(52.7%) | 5137(52.8%) | 0.711 |
|  | III-IV | 2322(47.0%) | 2261(47.3%) | 4583(47.2%) |  |

**Table S5.** The prevalence of post-treatment renal insufficiency (PTRI) and related parameters in patients with colorectal cancer

| Parameters | Percentage | Yes | No | Unknown |
| --- | --- | --- | --- | --- |
| eGFR decrease ≥ 25% | 3.9% | 405 | 10053 | 123 |
| Serum creatinine increase ≥ 25% | 4.4% | 458 | 10000 | 123 |
| Serum BUN increase ≥ 25% | 14.8% | 1557 | 8930 | 94 |
| Serum UA increase ≥ 25% | 0.2% | 17 | 10471 | 93 |
| Serum CysC increase ≥ 25% | 8.3% | 500 | 5529 | 4552 |
| Post-treatment renal insufficiency (PTRI) | 20.3% | 2132 | 8366 | 83 |

*eGFR, estimated glomerular filtration rate; BUN, blood urea nitrogen; UA, uric acid; CysC, cystatin C.

**Table S6.** The influence of treatment related parameters on prevalence of post-treatment renal insufficiency (PTRI) in patients with colorectal cancer

| Parameters |  | No PTRI  (N=5960) | PTRI (N=4496) | Total  (N=10456) | P |
| --- | --- | --- | --- | --- | --- |
| Radical resection | Radical | 4717(95.8%) | 1199(94.6%) | 5916(95.6%) | 0.073 |
|  | Palliative | 207(4.2%) | 68(5.4%) | 275(4.4%) |  |
| Laparoscopic surgery | Laparoscopic | 2982(60.4%) | 714(55.6%) | 3696(59.4%) | **0.002** |
|  | Open | 1957(39.6%) | 571(44.4%) | 2528(40.6%) |  |
| Extensive resection | No | 8178(97.8%) | 2083(97.7%) | 10261(97.7%) | 0.887 |
|  | Yes | 188(2.2%) | 49(2.3%) | 237(2.3%) |  |
| Postoperative complication | No | 4885(97.1%) | 1205(93.1%) | 6090(96.3%) | **<0.001** |
|  | Yes | 146(2.9%) | 89(6.9%) | 235(3.7%) |  |
| Intestinal ostomy | No | 2368(58.8%) | 531(52.8%) | 2899(57.6%) | **<0.001** |
|  | Yes | 1656(41.2%) | 475(47.2%) | 2131(42.4%) |  |
| Blood transfusion | No | 8254(98.7%) | 2067(97.0%) | 10321(98.3%) | **<0.001** |
|  | Yes | 112(1.3%) | 65(3.0%) | 177(1.7%) |  |
| Preoperative chemoradiotherapy | No | 4394(82%) | 1117(80.6%) | 5511(81.7%) | 0.224 |
|  | Yes | 964(18%) | 269(19.4%) | 1233(18.3%) |  |
| Preoperative radiotherapy | No | 4867(90.8%) | 1257(90.7%) | 6124(90.8%) | 0.869 |
|  | Yes | 491(9.2%) | 129(9.3%) | 620(9.2%) |  |
| Preoperative chemotherapy | No | 4454(83.1%) | 1136(81.9%) | 5590(82.9%) | 0.281 |
|  | Yes | 904(16.9%) | 251(18.1%) | 1155(17.1%) |  |
| Postoperative chemoradiotherapy | No | 1694(32.9%) | 397(29.9%) | 2091(32.3%) | **0.038** |
|  | Yes | 3458(67.1%) | 931(70.1%) | 4389(67.7%) |  |
| Postoperative radiotherapy | No | 4666(90.6%) | 1193(89.8%) | 5859(90.4%) | 0.419 |
|  | Yes | 486(9.4%) | 135(10.2%) | 621(9.6%) |  |
| Postoperative chemotherapy | No | 1708(33.2%) | 400(30.1%) | 2108(32.5%) | **0.035** |
|  | Yes | 3444(66.8%) | 928(69.9%) | 4372(67.5%) |  |
|  | | | | | |

**Table S7.** The univariate and multivariate logistics analysis of risk factors of post-treatment renal insufficiency in patients with colorectal cancer

| Parameters | Univariate analysis | | Multivariate analysis | |
| --- | --- | --- | --- | --- |
|  | HR (95%CI) | P | HR (95%CI) | P |
| Radical resection (palliative vs. radical) | 1.292(0.976-1.712) | 0.074 |  |  |
| Laparoscopic surgery (open vs. laparoscopic) | 1.219(1.077-1.379) | **0.002** | 1.252(1.089-1.440) | **0.002** |
| Extensive resection (yes vs. no) | 1.023(0.745-1.406) | 0.887 |  |  |
| Postoperative complication (yes vs. no) | 2.471(1.885-3.241) | **<0.001** | 2.398(1.653-3.477) | **<0.001** |
| Intestinal ostomy (yes vs. no) | 1.279(1.113-1.470) | **0.001** | 1.235(1.073-1.421) | **0.003** |
| Blood transfusion (yes vs. no) | 2.317(1.701-3.158) | **<0.001** | 1.303(0.762-2.227) | 0.333 |
| Preoperative chemoradiotherapy (yes vs. no) | 1.098(0.945-1.276) | 0.224 |  |  |
| Preoperative radiotherapy (yes vs. no) | 1.017(0.830-1.247) | 0.869 |  |  |
| Preoperative chemotherapy (yes vs. no) | 1.089(0.933-1.270) | 0.281 |  |  |
| Postoperative chemoradiotherapy (yes vs. no) | 2.149(1.708-3.310) | **<0.001** | 2.071(1.413-3.486) | **<0.001** |
| Postoperative radiotherapy (yes vs. no) | 1.086(0.889-1.328) | 0.419 |  |  |
| Postoperative chemotherapy (yes vs. no) | 1.151(1.010-1.311) | **0.036** | 1.501(0.091-2.746) | 0.426 |
|  | | | | |

**Table S8.** The univariate COX analysis of risk factors for disease-free survival and overall survival in patients with colorectal cancer.

| Parameters | Disease-free survival | | Overall survival | |
| --- | --- | --- | --- | --- |
|  | HR(95%CI) | P | HR(95%CI) | P |
| Sex (male vs. female) | 1.035(0.983-1.090) | 0.187 | 1.037(0.980-1.097) | 0.209 |
| Age (≥65 vs. <65year) | 0.971(0.879-1.073) | 0.564 | 1.005(0.902-1.120) | 0.925 |
| History of cancer (yes vs. no) | 1.624(1.276-2.067) | **<0.001** | 1.594(1.222-2.078) | **0.001** |
| Cardiovascular disease (yes vs. no) | 1.613(1.447-1.797) | **<0.001** | 1.592(1.415-1.792) | **<0.001** |
| Hypertension (yes vs. no) | 1.032(0.928-1.148) | 0.558 | 1.030(0.918-1.156) | 0.616 |
| Cerebrovascular disease (yes vs. no) | 1.408(0.975-2.032) | 0.068 | 1.429(0.962-2.122) | 0.077 |
| Diabetes (yes vs. no) | 0.990(0.858-1.142) | 0.886 | 0.976(0.834-1.141) | 0.758 |
| Respiratory disease (yes vs. no) | 1.724(1.208-2.461) | **0.003** | 1.724(1.170-2.542) | **0.006** |
| Self-reported CKD (yes vs. no) | 1.395(0.932-2.088) | 0.105 | 1.297(0.824-2.039) | 0.261 |
| CKD diagnosed by ultrasonography (yes vs. no) | 1.034(0.640-1.669) | 0.892 | 1.078(0.647-1.796) | 0.772 |
| Proteinuria (positive vs. negative) | 3.600(3.208-4.039) | **<0.001** | 3.582(3.158-4.063) | **<0.001** |
| Hematuria (positive vs. negative) | 1.764(1.574-1.977) | **<0.001** | 1.779(1.571-2.014) | **<0.001** |
| Baseline serum creatinine (elevated vs. normal) | 1.363(1.102-1.687) | **0.004** | 1.268(0.998-1.610) | 0.052 |
| Baseline eGFR (<60 vs. ≥60 mL/min/1.73m^2^) | 1.572(1.192-2.072) | **0.001** | 1.532(1.131-2.076) | **0.006** |
| Peak serum creatinine (elevated vs. normal) | 1.291(1.088-1.532) | **0.004** | 1.245(1.030-1.505) | **0.024** |
| Lowest eGFR (<60 vs. ≥60 mL/min/1.73m^2^) | 1.539(1.242-1.906) | **<0.001** | 1.454(1.145-1.847) | **0.002** |
| Baseline serum BUN (elevated vs. normal) | 1.813(1.480-2.223) | **<0.001** | 1.892(1.522-2.351) | **<0.001** |
| Peak serum BUN (elevated vs. normal) | 1.667(1.430-1.943) | **<0.001** | 1.749(1.484-2.060) | **<0.001** |
| Baseline serum UA (elevated vs. normal) | 1.114(0.949-1.308) | 0.186 | 1.027(0.858-1.230) | 0.769 |
| Peak serum UA (elevated vs. normal) | 1.136(0.970-1.330) | 0.114 | 1.049(0.879-1.253) | 0.597 |
| Baseline serum CysC (elevated vs. normal) | 1.079(0.924-1.261) | 0.338 | 0.978(0.820-1.166) | 0.801 |
| Peak serum CysC (elevated vs. normal) | 1.065(0.920-1.232) | 0.400 | 0.981(0.833-1.155) | 0.818 |
| Blood transfusion (yes vs. no) | 1.589(1.157-2.184) | **0.004** | 1.571(1.112-2.220) | **0.010** |
| Preoperative chemoradiotherapy (yes vs. no) | 0.848(0.735-0.978) | **0.023** | 0.903(0.775-1.052) | 0.191 |
| Preoperative radiotherapy (yes vs. no) | 0.800(0.653-0.980) | **0.031** | 0.885(0.715-1.094) | 0.259 |
| Preoperative chemotherapy (yes vs. no) | 0.850(0.733-0.984) | **0.030** | 0.915(0.783-1.071) | 0.269 |
| Preoperative targeted therapy (yes vs. no) | 1.480(1.124-1.948) | **0.005** | 1.450(1.069-1.968) | **0.017** |
| Preoperative immunotherapy (yes vs. no) | 0.613(0.153-2.455) | 0.490 | 0.728(0.182-2.916) | 0.654 |
| Tumor position (rectal vs. colon cancer) | 0.910(0.824-1.004) | 0.060 | 0.925(0.831-1.029) | 0.153 |
| Radical resection (palliative vs. radical) | 1.863(1.545-2.247) | **<0.001** | 1.690(1.366-2.092) | **<0.001** |
| Laparoscopic surgery (open vs. laparoscopic) | 1.015(0.907-1.137) | 0.790 | 0.974(0.861-1.102) | 0.676 |
| Extensive resection (yes vs. no) | 1.254(0.926-1.697) | 0.144 | 1.160(0.825-1.631) | 0.392 |
| Intestinal ostomy (yes vs. no) | 0.985(0.870-1.115) | 0.811 | 0.997(0.871-1.141) | 0.963 |
| TNM stage (III-IV vs. I-II) | 1.497(1.352-1.657) | **<0.001** | 1.571(1.406-1.756) | **<0.001** |
| Vascular invasion (positive vs. negative) | 2.354(2.121-2.613) | **<0.001** | 2.280(2.035-2.554) | **<0.001** |
| Perineural invasion (positive vs. negative) | 1.591(1.434-1.766) | **<0.001** | 1.624(1.451-1.819) | **<0.001** |
| Tumor budding (2-3 vs. 1) | 1.279(1.118-1.464) | **<0.001** | 1.276(1.102-1.478) | **0.001** |
| Tumor deposit (positive vs. negative) | 1.745(1.560-1.951) | **<0.001** | 1.699(1.503-1.920) | **<0.001** |
| dMMR (pMMR vs. dMMR) | 1.457(1.134-1.872) | **0.003** | 1.421(1.084-1.862) | **0.011** |
| KRAS (mutant vs. wild) | 1.033(0.928-1.150) | 0.553 | 1.014(0.902-1.140) | 0.818 |
| NRAS (mutant vs. wild) | 0.881(0.662-1.172) | 0.385 | 0.965(0.716-1.300) | 0.815 |
| BRAF (mutant vs. wild) | 1.456(1.126-1.882) | **0.004** | 1.400(1.054-1.859) | **0.020** |
| PIK3CA (mutant vs. wild) | 0.831(0.607-1.139) | 0.250 | 0.894(0.641-1.246) | 0.508 |
| Postoperative chemoradiotherapy (yes vs. no) | 1.233(1.094-1.390) | **0.001** | 1.243(1.091-1.417) | **0.001** |
| Postoperative radiotherapy (yes vs. no) | 1.286(1.091-1.516) | **0.003** | 1.253(1.046-1.502) | **0.015** |
| Postoperative chemotherapy (yes vs. no) | 1.232(1.093-1.388) | **0.001** | 1.239(1.088-1.411) | **0.001** |
| CEA (≥5 vs. <5 ng/mL) | 1.377(1.227-1.544) | **<0.001** | 1.407(1.242-1.593) | **<0.001** |
| CA199 (≥37 vs. <37 U/mL) | 1.524(1.324-1.753) | **<0.001** | 1.516(1.301-1.766) | **<0.001** |
| Proximal margin (positive vs. negative) | 0.636(0.090-4.517) | 0.651 | 0.747(0.105-5.302) | 0.770 |
| Distal margin (positive vs. negative) | 1.216(0.881-1.678) | 0.235 | 1.215(0.855-1.725) | 0.278 |
| Circumferential margin (positive vs. negative) | 1.723(1.336-2.222) | **<0.001** | 1.866(1.428-2.440) | **<0.001** |
| Differentiation (poor vs. well or moderate) | 1.453(1.282-1.647) | **<0.001** | 1.400(1.220-1.607) | **<0.001** |
| Baseline renal insufficiency (RI, yes vs. no) | 2.262(2.049-2.498) | **<0.001** | 2.122(1.905-2.362) | **<0.001** |
| Post-treatment renal insufficiency (PTRI, yes vs. no) | 1.448(1.293-1.622) | **<0.001** | 1.446(1.279-1.636) | **<0.001** |
| Tumor invasion of adjacent organs (T4b, yes vs. no) | 1.826(1.703-1.985) | **<0.001** | 1.803(1.612-2.013) | **<0.001** |

*CKD, chronic kidney disease; eGFR, estimated glomerular filtration rate; BUN, blood urea nitrogen; UA, uric acid; CysC, cystatin C.

**Table S9.** Multivariate COX analysis of risk factors for disease-free survival and overall survival in colorectal cancer patients without pre-existing chronic kidney disease

| Parameters | Disease-free survival | | Overall survival | |
| --- | --- | --- | --- | --- |
|  | HR(95%CI) | P | HR(95%CI) | P |
| Preoperative baseline renal insufficiency (RI, yes vs. no) | 1.778(1.581-1.999) | **<0.001** | 1.640(1.442-1.866) | **<0.001** |
| Post-treatment renal insufficiency (PTRI, yes vs. no) | 1.293(1.144-1.461) | **<0.001** | 1.329(1.177-1.501) | **<0.001** |
| Radical resection (palliative vs. radical) | 1.656(1.362-2.013) | **<0.001** | 1.520(1.217-1.898) | **<0.001** |
| TNM stage (III-IV vs. I-II) | 1.283(1.135-1.450) | **<0.001** | 1.344(1.176-1.537) | **<0.001** |
| Vascular invasion (positive vs. negative) | 2.330(2.057-2.641) | **<0.001** | 2.218(1.935-2.544) | **<0.001** |
| Perineural invasion (positive vs. negative) | 1.209(1.066-1.372) | **0.003** | 1.292(1.126-1.482) | **<0.001** |
| Tumor deposits (positive vs. negative) | 1.230(1.075-1.407) | **0.003** | 1.197(1.033-1.387) | **0.017** |
